# Progress in development of bioderived materials for dermal wound healing

**DOI:** 10.1093/rb/rbx025

**Published:** 2017-10-09

**Authors:** Lin-Cui Da, Yi-Zhou Huang, Hui-Qi Xie

**Affiliations:** * Laboratory of Stem Cell and Tissue Engineering, State Key Laboratory of Biotherapy, West China Hospital, Sichuan University, Chengdu, Sichuan 610041, People’s Republic of China

**Keywords:** wound healing, tissue-engineered skin, bioderived materials, acellular matrix

## Abstract

Treatment of acute and chronic wounds is one of the primary challenges faced by doctors. Bioderived materials have significant potential clinical value in tissue injury treatment and defect reconstruction. Various strategies, including drug loading, addition of metallic element(s), cross-linking and combining two or more distinct types of materials with complementary features, have been used to synthesize more suitable materials for wound healing. In this review, we describe the recent developments made in the processing of bioderived materials employed for cutaneous wound healing, including newly developed materials such as keratin and soy protein. The focus was on the key properties of the bioderived materials that have shown great promise in improving wound healing, restoration and reconstruction. With their good biocompatibility, nontoxic catabolites, microinflammation characteristics, as well as their ability to induce tissue regeneration and reparation, the bioderived materials have great potential for skin tissue repair.

## Introduction

**Figure 1.**
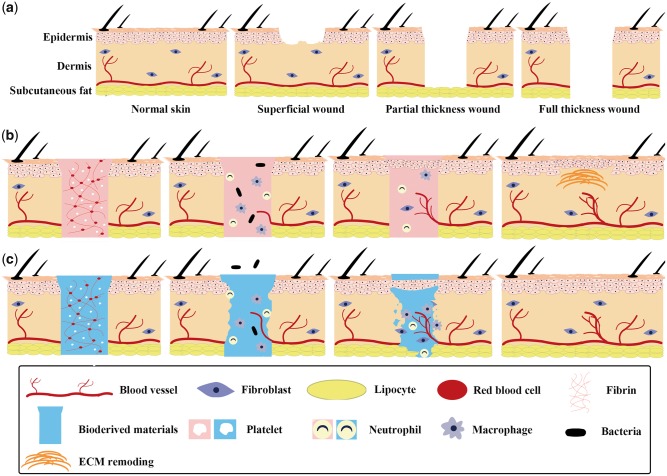
Diagrammatic sketch of wounds and wound healing. (**a**) classification of skin wounds based on depth. (**b**) schematic illustration of classical healing process of full-thickness wounds. (**c**) schematic illustration showing healing of full-thickness wounds using bioderived materials

The skin, which is composed of the epidermis, the dermis and subcutaneous tissue (Fig. 1a), plays a critical role in ensuring human health, for it protects tissues and organs from physical, mechanical, chemical and microbial damage [1]. Wounds of the skin can be classified as superficial, partial-thickness and full-thickness wounds (Fig. 1a) based on their thickness [2]. Although most dermal wounds are healed by a natural healing process (Fig. 1b), the healing process may be limited or may fail to regenerate and reconstruct fully functional skin tissue under acute or severe conditions, as in the case of partial-thickness and full-thickness wounds [3]. Therefore, skin wound healing, especially under acute or severe conditions, is a common challenge encountered by plastic and reconstructive surgeons. The use of bioderived materials, which are prepared from specially treated naturally occurring tissues, has become an attractive approach for treating such wounds because of the ability of these materials to inhibit bacterial growth, stimulate cell growth and angiogenesis, and enhance wound healing [3–6]. Figure 1c shows a diagrammatic sketch of wound healing using bioderived materials.

However, their clinical significance is limited because of their unsatisfactory mechanical properties, biodegradability and reproducibility. In this regard, various strategies have been developed in recent years to address these problems and synthesize more suitable materials for wound healing. These advanced strategies include drug loading, adding elements (e.g. gold, silver and zinc, among others), cross-linking and combining two or more diverse types of materials with complementary features. This article aims to review the most relevant recent developments in the synthesis of bioderived materials for cutaneous wound healing, including newly reported materials such as keratin and soy protein. The key properties of the bioderived materials that have shown great promise in improving wound healing, restoration and reconstruction are discussed.

## Scaffolds from natural polymers

**Figure 2.**
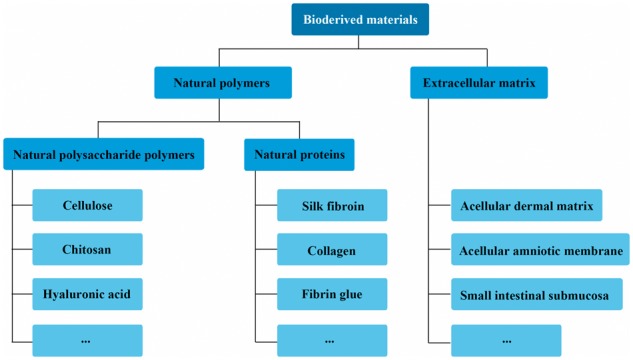
Classification of bioderived materials based on chemical composition of scaffolds

The use of natural polymers, including natural polysaccharide polymers (e.g. cellulose, chitosan and hyaluronic acid, among others) and natural proteins (e.g. silk fibroin, collagen and fibrin glue, among others) (see Fig. 2), is an important direction in the development of tissue engineering scaffolds, because these materials exhibit high cellular affinity, and their use does not result in chronic inflammation, immunological reactions, or toxicity [7].

### Natural polysaccharide polymers

#### Cellulose

Cellulose, which is one of the most abundant natural polysaccharides, has attracted considerable interest because of its high durability and low inflammatory response. Further, bacterial cellulose (BC), which is generated through the bacterial fermentation of glucose, is being explored extensively. Its advantages, such as its high wetting strength, elasticity and permeability as well as its ability to accelerate wound healing and reduce scar tissue, make it highly suited for the healing of skin wounds. It exhibits linear elastic behaviour for elongations greater than 35%, with its tensile properties being similar to that of the porcine carotid artery [8]. Different nanoscale structures can have different effects on skin wound healing. In general, BC films with larger pores and a looser and rougher structure result in better cell migration, a higher recovery rate and weaker inflammatory response [9].

However, BC is synthesized by bacteria and exhibits a certain degree of immunogenicity and low antibacterial activity, which limit its applicability. Its antibacterial activity can be improved by loading it with a suitable drug or by adding various elements to it (e.g. silver and copper), while its immunogenicity can be reduced through chemical modification [10, 11]. Another shortcoming may be the difficulty in forming BC in the desired geometries. Bottan *et al.* could obtain nanofibers with the desired geometrical characteristics through guided assembly-based biolithography (GAB) and were thus able to effectively control the cellular activities that are fundamental to skin wound healing [12].

#### Chitosan

Chitosan (CS), a deacetylated form of chitin whose structure is similar to that of glycosaminoglycans (GAG), has promising prospects in wound repair [13–16]. Upon depolymerization caused by enzymes such as glucosaminidases, lipases and lysozyme, chitosan yields bioactive chito-oligosaccharides, which show superior antimicrobial properties, and N-acetyl-b-d-glucosamine (NAG), which can promote hyaluronic acid synthesis and fibroblast proliferation and facilitate ordered collagen deposition at the wound site [13, 17].

Several factors affect wound healing, including the molecular weight of the CS sample used, the degree of deacetylation, which is the molar ratio of the d-glucosamine units to the sum of the NAG and d-glucosamine units, and the physiochemical modifications made [18, 19]. In general, the higher the molecular weight and degree of deacetylation of the CS sample used, the higher the wound-healing rate will be [20].

CS is a basic polysaccharide and although the free amino groups of CS can be protonated under acidic conditions, thus making it water soluble, its applicability remained limited because of its poor solubility under the pH conditions of the body and because of its loose cationic nature [21, 22]. Strategies to address these problems include the modification of CS by combining it with metal (oxide) nanoparticles (such as those of ZnO and Ag, see Table 1). The CS derivatives reported in the literature in the last few years are N-succinyl-chitosan, Oleoyl CS, quaternised CS, O-carboxymethyl CS and N,N,N-trimethyl-chitosan [23–29]. However, impurities such as the myosin residues generated during the extraction of CS can lead to an inflammatory reaction and/or the formation of microabscesses after transplantation [30]. Accordingly, high-purity CS should be used in skin tissue engineering.

**Table 1.** Studies (2012–17) on repairing skin tissue using natural polysaccharide polymers

Material	Fabrication method	Biological *in vitro* results	Biological *in vivo* results	Reference
Bacterial cellulose	Guided assembly-based biolithography	HDF seeded on BC for 12 h; immunostaining and wide-field microscopy: higher cell density was measured and specific cell alignment was detected on structured BC, in contrast to flat control substrates.	BC dressing on artificial wounds in live mouse model for 21 days: structured BC was not degraded or invaded by host cells; better fibroblast infiltration and new collagen deposition in structured BC with levels of vascularization and inflammation comparable to those elicited at interface with autologous full skin graft.	[12]
	Vaccarin solution impregnation	Cell viability studies: L929 cells incubated in BC extract for 24 and 72 h; MTT assay: after 24 h incubation, cell viability of BC membranes = 121.9%; cell viability of BC–Vac membranes =137.5%; after 72 h incubation, cell viability of BC membranes = 73.6%; cell viability of BC–Vac membranes = 80.7%; Cell attachment studies: L929 cells seeded on BC membrane membranes for 24 h; SEM: live cells exhibited better morphology on BC–Vac membranes.	BC dressing on artificial wounds in live mouse model for 14 days: new neovascularization, stratified squamous epithelium, dense new-born subcutaneous tissue, collagen fibre and hyperplastic fibrous connective tissue were observed in BC- and BC–Vac-membrane-treated groups.	[41]
	ZnO nanoparticle suspension impregnation	Samples placed over agar plates containing lawn of selected bacterial strains and incubated at 37 °C for 24 h; antimicrobial assay: BC–ZnO nanocomposites showed activity against gram-negative bacterial strains, including *Escherichia coli* (90%), *Citrobacter freundii* (90.9%) and *Pseudomonas aeruginosa* (87.4%) and gram-positive bacterial strain *Staphylococcus aureus* (94.3%).	BC–ZnO nanocomposites on artificial wounds in live mouse model for 15 days: healthy granulated tissue, regenerated sebaceous glands and new blood vessels and epithelium in regeneration were observed in BC–ZnO-nanocomposite-treated group; in contrast, ulceration and necrotic tissues were observed in negative group.	[42]
Chitosan	Introducing succinyl groups into glucosamine units of CS N-terminal	Cytotoxicity assay: L929 cells incubated in NSC or CS extract for 72 h; MTT assay: cell viability of NSC = 130% > cell viability of CS (*P* < 0.01) Antimicrobial activity: aqueous solution of NSC had superior antibacterial effects against *S. aureus* and *E. coli* compared to CS.	NSC powder or CS powder on artificial wounds in live rabbit model for 14 days: rate of macroscopic wound healing: NSC > CS > control; NSC-treated wounds showed better-organized superficial epithelium and were nearly completely repaired, with more fibroblasts, neovascularization and collagen tissues, as well as clearer and orderly boundary layer between epidermis and dermis.	[23]
	Mixing with hexagonal nanoparticles	Cytotoxicity assay: peripheral blood mononuclear cells, keratinocytes, or fibroblasts seeded on CS for 24 h; MTT assay: both keratinocytes and fibroblasts exhibit normal or moderately enhanced growth on CS films containing hexagonal nanoparticles.	—	[40]
Hyaluronic acid	Anti-TNF-α conjugation	TNF-α capture: conjugate or antibody applied on surface of collagen gel for 15, 30, 60 and 90 min; ELISA analysis: both (anti-TNF-α)–HA and anti-TNF-α result in capturing nearly 90% of TNF-α in collagen gel within 90 min, with slower capture by (anti-TNF-α)–HA than by anti-TNF-α over first 60 min.	Anti-TNF-α in PBS, (anti-TNF-α)–HA on artificial burn wounds in adult Sprague–Dawley rat models for 24 h: at 24 h, more antibodies are present than would be expected in first 600 mm of wound. Below 100 mm from wound surface is cell-dense area that corresponds to first peak of antibody accumulation at both 1 and 24 h.	[43]
	Mixing with PFC	—	HA scaffolds containing PFC on left facial wound in 52-year-old man for 3 weeks: healthy granulation tissue and periulcer epithelization were noted. Remaining ulcer epithelized within several weeks, and no recurrence was observed as of 1-year follow-up. HA scaffolds containing PFC on left hand wound in 61-year-old woman for 2 weeks: wound had almost completely closed, whereas similar wound on fourth finger healed with standard conservative treatment but left hypertrophic scar.	[44]

HDF, human dermal fibroblasts; Vac, vaccarin; NSC, CS scaffold after introduction of succinyl groups into the glucosamine units of the N-terminal; ELISA, enzyme-linked immunosorbent assay; PFC, platelet-derived factor concentrate.

#### Hyaluronic acid

Hyaluronic acid (HA), which is distributed widely in tissues such as the skin, cardiac valves and the umbilical cord, is an anionic glycosaminoglycan with good hydrophilicity, viscoelasticity and lubrication [31–33]. As an essential component of the vertebrate extracellular matrix (ECM), HA is not only free of immunogenicity but also provides a suitable growth environment for cells and regulates cell adhesion, migration, proliferation and differentiation [34]. It can reduce graft contracture during skin healing by stimulating the production of more CD44 receptors, which advance the local enzymolysis of HA and promote wound surface vascularization. After the addition of HA during skin healing, the expressions of collagen I and III increase, while the ratio of the two collagen types decreases [35]. Further, in contrast to high-molecular-weight HA, low-molecular-weight HA promotes angiogenesis and granulated tissue formation [36].

The use of HA for scaffolds, however, is restricted because it readily dissolves and biodegrades in the body. Thus, the modification of HA using various cross-linking methods, such chemical or photoinduced cross-linking, is usually necessary. The use of cross-linked HA networks in rats, dogs and horses has yielded positive results, especially with respect to the healing rate [37–39].

In the last few years (2012–2017), many research groups have studied the possibility of using natural polysaccharide polymers that have been modified with bioactive substances or nanoparticles to improve their cell viability and antibacterial activity and strengthen their effect on tissue formation for skin tissue repair (Table 1). Further, CS films with hexagonal silver nanoparticles that generate heat and exhibit enhanced drug delivery properties when illuminated by infrared light have been synthesized (Table 1) [40]. These materials were found to be suitable for healing dermal wounds because they not only facilitated cell proliferation and mitigated bacterial infections but also promoted the delivery of small molecules into the cells.

### Natural proteins

#### Silk fibroin

Silk fibroin (SF) is a natural macromolecular protein consisting of both light and heavy chains, which are linked by a single disulphide bond, in a 1:1 ratio [45]. SF is suitable for developing wound-healing materials because of its desirable properties, including its high biodegradability, ease of chemical modification, good oxygen and water vapor permeabilities, and ability to provide a moist environment for cells [46, 47]. In addition, SF scaffolds with nanosized fibres are better suited for cell adhesion and the spreading of collagen I [47].

The molecular weight of the SF sample used may affect wound healing [46]. In general, SF with a narrow molecular-weight distribution heals faster with more re-epithelization and results in smaller residual scars and fewer infections and foreign body reactions than does SF with a wider molecular-weight distribution.

However, tailoring the mechanical properties of SF to the desired level remains a challenge for researchers because most of the biomaterials developed using SF solutions are weak and brittle, unlike native silk fibres [48].

#### Fibrin glue

Fibrinogen extracted by methods such as ammonium sulfate precipitation, polyethylene glycol precipitation, or ethanol precipitation is treated with thrombin to fabricate fibrin glue (FG), whose structure and mechanical strength can be regulated by varying the degree of cross-linking [49, 50]. FG exhibits various desirable biological properties and is a promising choice for tissue engineering scaffolds as well as a bioactive substance carrier [51, 52].

FG can improve haemostasis, reduce bacterial infections, help fibroblasts in the surrounding tissue to move through the wound, and increase the rate of keratinocyte spreading and replication [53]. Evidence shows that FG expresses the basal keratin-14 gene and the collagen I gene and forms a fully autologous skin construct with a continuous epidermal–dermal junction similar in structure to native skin *in vivo* [54]. FG is being used commercially and has been found to be superior in all aspects (including with respect to exudation, coloration, oedema and cosmetic appearance) as compared to platelet-rich plasma during clinical evaluations [53, 55]. However, the shortcomings of FG with respect to wound healing include its high cost, difficulty in storage, long preparation time and the potential risk of transfusion-transmitted diseases [53].

#### Collagen

Collagens, which are a family of fibrous proteins consisting of α-chains with a triple-helical structure, are the major constituents of the healthy dermal ECM and play a primary role in regulating tissue remodelling during wound healing [56–58]. Collagen can provide cell-binding sites such as those for integrin receptors to regulate cell adhesion, migration and other cellular processes, including proliferation and differentiation in the early stage [59]. It also offers several other advantages. For instance, it can be readily isolated and purified, exhibits low toxicity, and its mechanical, chemical and immunological properties are well documented [60, 61].

However, the journey from bench to bedside is a long one. Hence, more attention should be paid not only to the construction of scaffolds with optimized structures and properties but also to the development of collagen-like materials that mimic natural collagen in terms of their properties [62].

Several other natural polysaccharide polymers and natural proteins, such as starch, pectin, the carbohydrate polymers produced by bacteria and fungi, alginate, keratin, wool, gelatin and soy protein, can be used for skin wound healing [21, 63–68]. Recent relevant studies (2012–2017) on natural proteins for skin tissue repair are listed in Table 2. However, despite their excellent biocompatibility, the suitability of natural proteins for use in tissue engineering applications will increase only if their mechanical properties, biodegradability and reproducibility can be improved to match the application requirements.

**Table 2.** Studies (2012–17) on repairing skin tissue using natural proteins

Material	Fabrication method	Biological *in vitro* results	Biological *in vivo* results	Reference
Silk fibroin	Mixing with SSD	NHEK and NHEF separately seeded on SF–SSDX for 1 h; microscope: a significantly (*P* < 0.05) higher number of NHEK adhered to SF than to SF–SSDX. Initial spreading of NHEK adhering to SF–SSDX decreased gradually when SSD concentrations were increased. The case for NHEF was the same.	SF–SSDX on artificial wounds in live rat model for 14 days: sizes of wounds treated with SF/AgS 1.0 and Acticoat were much smaller than those in other groups, and rate of closure of wound treated with SF–SSDX was significantly higher (*P < *0.05) than that in control group.	[69]
	Immobilization with Cys-KR12	HaCaT seeded on Cys-KR12-immobilized SF for 10 days; Western blot: expression of involucrin increased in Cys-KR12-immobilized SF group. Raw264.7 seeded on Cys-KR12-immobilized SF for 12 h and stimulated with LPS for 6 h; ELISA: TNF-α expression of Raw264.7 cells was significantly more repressed on Cys-KR12-immobilized SF group than in the case of other groups after LPS stimulation.	—	[70]
Fibrin glue	Cryoprecipitation and cryocentrifugation	—	FG grafting on artificial wounds in live pig model for 14 days: number of new microvessels was significantly higher (*P* < 0.05) in FG group than in control group at day 3. Intensity of inflammation was significantly lower in FG group than in control group at day 7.	[71]
Collagen	Incorporation with gold nanoparticles	Cytotoxicity assay: L929 cells incubated in CS–AuX extract for 24 h; MTT cell viability assay: cell viability for all scaffold extracts was higher than 90%. Cell attachment assay: L929 fibroblasts seeded on CS–AuX for 24 h; SEM: fibroblasts on scaffolds gained their natural spindle-like shape with outstretched pseudopods spreading over the surface.	CS–AuX on artificial wounds in live rat model for 14 days: milder inflammatory reaction and higher neovascularization were observed with CS–AuX than in other groups; better wound closure was observed with CS–X, CS–AuX and MatriDerm than in untreated control.	[72]
	Modification with CBD-E7 peptide	—	Collagen/CBD-E7 peptide on artificial wounds in live porcine model for 28 days: significantly more MSCs were retained on CBD-E7 collagen scaffold than on pristine collagen scaffold at day 3 post-surgery; significantly rapid wound healing in collagen/CBD-E7 peptide group at days 14, 21 and 28 than in other groups; significantly higher capillary density in collagen/CBD-E7 peptide group than in other groups.	[73]

SSD, C_10_H_9_AgN_4_O_2_S; NHEK, normal human epidermal keratinocyte; NHEF, normal human epidermal fibroblast; HaCaT, human keratinocytes; LPS, lipopolysaccharides; Raw264.7, murine monocytes; CS–AuX, collagen-containing gold nanoparticles; MSCs, mesenchymal stem cells.

## Scaffolds from extracellular matrix

Cutaneous wound healing is a complex process requiring the integration of biological and molecular events that include cell migration and proliferation, ECM deposition, angiogenesis and remodelling [74]. ECM scaffolds can consist of a diverse range of molecules (e.g. collagen, fibrin, elastin, glycosaminoglycans and growth factors, among others) that mediate structural and/or biological properties [75]. They find wide use in tissue engineering owing to their inherent advantages such as their excellent biocompatibility, desirable biological activity, and ability to promote tissue regeneration. As a result, they have recently become the focus of growing interest [7, 75, 76]. In addition, they can release growth factors in a highly spatiotemporally controlled manner and can modulate their intracellular signalling [59]. A number of studies have shown that ECM scaffolds from one type of tissue can provide abundant growth signals for the cells of another type of tissue [77, 78]. In the field of skin tissue engineering, the acellular dermal matrix (ADM), acellular amniotic membrane (AAM) and small intestinal submucosa (SIS) have been studied extensively.

The ADM is the remaining ECM of the dermal and basement membranes once the cellular components (the epidermis and the cells of the dermis) have been removed and can provide the mechanical support lacking at the wound site, improve the quality of wound healing, increase the survival rate of the epidermal membrane, and prevent wound contraction and scar formation [79]. Dermal grafts obtained from human subjects have been used to treat burns and full-thickness skin defects [80, 81]. However, their applicability is limited by their high cost, the limited availability of cadaver skin, and the risk of disease transmission [82]. Thus, ADMs derived from the skins of pigs, goats, fish and ostriches have been used; these have yielded satisfactory results with respect to wound healing [83–85]. The stiffness of the ADM may affect wound healing. In general, epidermis treated with a softer ADM derived from younger mice exhibits better collagen density than that treat with ADM derived from older mice. In addition, the orientation and stiffness of the new dermal tissue grown closer to the normal tissue are also favourable [86].

The AAM, which is composed of collagen, elastin, laminin, fibronectin and growth factors, is an attractive biomaterial for use in wound healing, as it helps reduce pain and wound dehydration, promotes epithelialization, prevents scarring and exhibits anti-inflammatory, antimicrobial and antifibroblastic effects [87–89]. AAMs prepared by the chemical detergent-enzymatic extraction method have been shown to preserve the tissue matrix and the reticular structure well and thus have promise for use as membranes for skin wound healing [90].

SIS, an ECM material derived from swine, is more advantageous for promoting angiogenesis, cell growth and differentiation and tissue regeneration because of its innate growth factors (transforming growth factor-beta, basic fibroblast growth factor, vascular endothelial growth factor and epidermal growth factor, among others) [91]. It also exhibits several advantages, such as immunogenicity, good cellular compatibility and anisotropy and therefore shows promise for use in the repair of soft tissue [92–95]. In addition, seeding MSCs on SIS can promote appendage formation with less scarring [96].

Recent relevant studies (2012–2017) on ECM for skin tissue repair are listed in Table 3.

**Table 3.** Studies (2012–17) on repairing skin tissue using extracellular matrix

Material	Fabrication method	Biological *in vitro* results	Biological *in vivo* results	Reference
Acellular dermal matrix	Seeding with BMSCs	BMSCs seeded on ADM for 14 days; confocal microscopy: proliferation indexes of MSC in ADM scaffolds on days 1, 4, 7 and 14 were 0.18 ± 0.07%, 0.32 ± 0.04%, 0.45 ± 0.11% and 100 ± 0.09%, respectively.	ADM on artificial wounds in live mice model for 21 days: dermal differentiation, epithelial maturation, skin appendage regeneration and neovascularization of wound treated with BMSC-seeded ADM scaffolds were better than those in control group.	[97]
	PHD-2 siRNA solution impregnation	—	ADM on artificial wounds in live mice model for 14 days: cellularity and vascularity of wound treated with PHD-2 siRNA–ADM scaffolds were better than those in control group.	[98]
Acellular amniotic membrane	Seeding with ADMSCs	ADMSCs seeded on AAM for 7 days; HE staining: spindle-like ADMSCs grew well at day 3; ADMSCs fused into patches on surface of AAM and turned into multiple layers at day 7.	AAM on artificial wounds in live nude mice model for 28 days: wound-healing rate and number of epidermal layers in ADMSC–AAM seeding group were significantly higher than those in hAM and AAM groups (*P* < 0.05); typical hair follicle structure appeared in ADMSC–AAM seeding group.	[99]
Small intestinal submucosa	Seeding with BMSCs	BMSCs seeded on SIS for 21 days; MTT assay and HE staining: BMSCs grow and proliferate well on SIS scaffolds.	SIS on artificial wounds in live rat model for 7 weeks: epithelization in decellularized SIS group was faster than in native SIS; skin appendage-like structures were observed only in decellularized SIS group at day 28.	[100]

BMSCs, bone marrow mesenchymal stem cells; PHD2, prolyl hydroxylase domain-2; ADMSCs, adipose-derived mesenchymal stem cells.

## Scaffolds from composite biomaterials

Tissues and organs are complex and orderly wholes composed of many different components. The ideal scaffold must exhibit certain properties, particularly high biocompatibility, bioactivity and mechanical strength.

**Figure 3.**
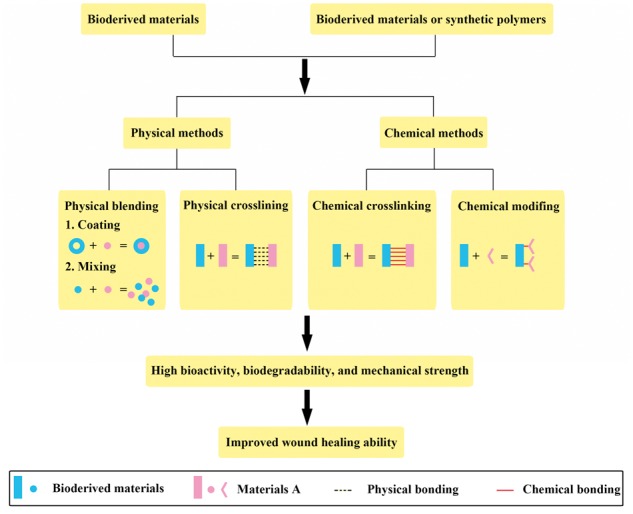
Classification of methods for fabricating composite scaffolds

Recent research has focused on combining two or more different materials with complementary features using physical or chemical methods to fabricate scaffold materials. These composites exhibit improved performance as dermal wound-healing materials (Table 4). Composites developed using several natural polymers can yield scaffolds similar to the ECM [101]. In addition, composites developed from synthetic polymers and bioderived materials can endow materials with various desirable properties such as high bioactivity, biodegradability and mechanical strength (Fig. 3) [102–104].

**Table 4.** Studies (2012–17) on repairing skin tissue using composite biomaterials

Classification	Material	Fabrication method	Biological *in vitro* results	Biological *in vivo* results	Reference
Natural polymers–natural polymers	Gelatin–HA	Electrospinning and cross-linking	—	Gelatin–HA on artificial wounds in live rat model for 14 days: more epidermis and fewer inflammatory cells were found in GE/HA nanofiber and ChitoHeal gel groups than in antiseptic gauze group.	[105]
Natural polymers–ECM	Decellularized peritoneum– HA–EGF	1. Coating decellularized peritoneum with sodium hyaluronate 2. Soaking in EGF solution	NIH3T3 cells cultured in culture medium containing EGF for 96 h; MTT assay: cell viability increased as EGF concentrations increased.	Scaffolds on artificial wounds in live rabbit model for 20 days: decellularized peritoneum–HA–EGF group recovered best among all groups, with wound-healing rates of 87.41% after 20 days post-surgery; thicker epidermis and dermis layers were observed in decellularized peritoneum–HA–EGF group than in decellularized peritoneum group.	[106]
Natural polymers–synthetic polymers	PCL–CA–CS–collagen	1. Co-electrospinning of PCL and CA 2. Alternately soaking in CS and collagen solutions every 20 min	Cytotoxicity assay: NHDFs seeded on scaffolds for 72 and 120 h; MTT assay, flow cytometry analysis and ultrastructure: cell viability and cell density in PCL–CA–CS–collagen group were higher than those in other groups; mitochondria in cells with cytoplasmic vacuolization appeared to be normal with increase in number of CS/collagen bilayer coatings on PCL–CS mats. Cell migration assay: NHDFs seeded on scaffolds for 7 days; optical microscopy: NHDF migration into wound area of PCL–CA–CS–collagen was greatly enhanced with increase in number of bilayer coatings.	Fibroblast-seeded scaffolds on artificial wounds in live rat model for 7 days: the CS/collagen coatings in scaffolds had positive effect on neovascularization and led to increased wound-healing rate; fibroblast-seeded PCL–CA–CS–collagen promoted complete re-epithelialization and regeneration of skin appendages; regenerated skin with fibroblast-seeded PCL–CA–CS–collagen covering exhibited smooth surface and loose collagen fibre arrangement similar to that of normal skin.	[107]
	Castor-oil-based polymer–CS–ZnO	1. Mixing castor oil with CS–ZnO nanoparticles 2. Reacting with HDI 3. Crosslinking using GLA	Cell viability study: NHDF incubated in bionanocomposites for 72 h; alamarBlue assay: castor-oil-based polymer–CS–ZnO whose CS–ZnO loading rate ≤ 5.0 wt% showed no toxic effects. Antimicrobial assay: castor-oil-based polymer–CS–ZnO exhibited antimicrobial activity against *S. aureus*, *Micrococcus luteus* and *E. coli*, with the effect increasing with CS–ZnO concentration.	Castor-oil-based polymer–CS–ZnO on artificial wounds in live rat model for 14 days: castor-oil-based polymer–CS–ZnO group healed much faster with better re-epithelialization and collagen deposition than did castor oil group and gauze group.	[108]

EGF, epidermal growth factor; PCL, polycaprolactone; CA, cellulose acetate; NHDFs, normal human dermal fibroblast; HDI, hexamethylene diisocyanate; GLA, glutaraldehyde.

## Challenges and perspectives

Owing to their unique properties, bioderived materials have significant clinical value in facilitating and accelerating wound healing and inducing skin regeneration in full-thickness wounds. However, their potential for use in dermal wound healing would increase further if their mechanical properties, biodegradability and reproducibility could be improved to fit the application requirements better. These limitations may be addressed through cross-linking, combining two or more different types of materials with complementary features, or by developing other novel strategies in the future.
